# Two novel mutations identified in ADCC families impair crystallin protein distribution and induce apoptosis in human lens epithelial cells

**DOI:** 10.1038/s41598-017-18222-z

**Published:** 2017-12-19

**Authors:** Li Li, Da-Bei Fan, Ya-Ting Zhao, Yun Li, De-Qian Kong, Fang-Fei Cai, Guang-Ying Zheng

**Affiliations:** 1 Ophthalmologic Center, the First Affiliated Hospital of Zhengzhou University, Zhengzhou, 450052 China; 2 Henan Provincial Eye Hospital, Zhengzhou, Henan 450052 China; 3 Endocrine Department, the First Affiliated Hospital of Zhengzhou University, Zhengzhou, 450052 China

## Abstract

Congenital cataract (CC) is a clinical and genetically heterogeneous eye disease that primarily causes lens disorder and even amblyopic blindness in children. As the mechanism underlying CC is genetically inherited, identification of CC-associated gene mutations and their role in protein distribution are topics of both pharmacological and biological research. Through physical and ophthalmic examinations, two Chinese pedigrees with autosomal dominant congenital cataract (ADCC) were recruited for this study. Mutation analyses of CC candidate genes by next-generation sequencing (NGS) and Sanger sequencing revealed a novel missense mutation in CRYBB2 (p.V146L) and a deletion mutation in CRYAA (p.116_118del). Both mutations fully co-segregated were not observed in unaffected family members or in 100 unrelated healthy controls. The CRYBB2 missense mutation disrupts the distribution of CRYBB2 in human lens epithelial cells (HLEpiCs), and the CRYAA deletion mutation causes hyperdispersion of CRYAA. Furthermore, these two crystallin mutations result in aberrant expression of unfolded protein response (UPR) marker genes as well as apoptosis in HLEpiCs. Collectively, these findings broaden the genetic spectrum of ADCC.

## Introduction

Congenital cataract (CC) is a major cause of infant blindness and remains a significant health-care burden in children worldwide^1,2^. CC is characterized by impaired and abnormal expression of crystallin, resulting in lens protein aggregation, which blocks light as it passes through the lens^3,4^. Globally, nearly 0.01–0.15% of newborns suffer from CC. One-third of cases are inherited, and despite reports of a few cases of autosomal recessive and x-linked inheritance, the vast majority of CC is attributed to autosomal dominant inheritance with high clinical and genetic heterogeneity^4–6^. To date, more than 20 genes have been identified as being responsible for autosomal dominant cataracts; among these, crystallin genes are the most common cause of CC, accounting for 50% of autosomal dominant cataracts^7^. Crystallin proteins can be divided into two categories based on their characteristics: α-crystallins and β/γ-crystallins. The most abundant soluble protein in the lens, α-crystallin prevents lens cell apoptosis and protects protein stability; α-crystallin can be further divided into two sub-classes, αA- and αB-crystallin, which are encoded by *CRYAA* and *CRYAB*, respectively^8^. Some missense mutations in the *CRYAA* gene that result in the substitution of an arginine with a neutral or hydrophobic amino acid occur in the core domain of α-crystallin^9–15^, and several missense mutations sites in *CRYAA* have been linked to CC. These mutations may result in the loss of the α-crystallin protein, leading to increased light scattering and lens opacification^16,17^. Predominantly structural protein, β-crystallin contain four key Greek motifs and are involved in lens development and the maintenance of lens transparency^18^. Although various CC-causing mutations have been reported in *CRYAB*, mutations in the key Greek motifs appear to enhance protein-protein interactions or aggregation and protein denaturation, ultimately leading to cataracts^19–22^. Despite the association of numerous mutations with cataracts, missense mutations in crystallin genes, particularly *CRYAA* and *CRYBB2*, are considered the primary cause of autosomal dominant cataracts. Indeed, according to *Cat-Map* statistics, approximately 70% of autosomal dominant cataracts may be related to missense mutations in crystallin genes^23^. Previous studies have reported that the apoptosis triggered by cataract-related mutant proteins is a result of the unfolded protein response (UPR). UPR, which is caused by unfold protein or oxidative damage, comprises a set of intracellular signaling pathways that were recently reported to be activated in the lens during development and endoplasmic reticulum stress^24,25^. For example, Ma *et al*. found that splicing mutations in the human βA3/A1-crystallin gene *CRYBA1* result in severe misfolding of the protein and activate the UPR stress pathway and eventually apoptosis^26^. In keeping with these findings, the R49C missense mutation in αA-crystallin is related to upregulation of the PERK UPR pathway in the mouse lens, ultimately leading to apoptosis^16^, and variable activation of UPR is observed with the Cx50 mutant (S50P, G22R) in mice^27^. Moreover, induction of UPR with successive apoptosis in lens epithelial cells is expected to be involved in CC formation^28^.

Regardless, there is no consensus regarding the role of crystallin mutations that result in apoptosis or its molecular mechanism in CC development. In this study, we performed genetic analysis in an attempt to identify causative genes in two Chinese families affected by autosomal dominant congenital cataract (ADCC) through next-generation sequencing (NGS) and Sanger sequencing. Two novel mutations, including one missense mutation in *CRYBB2* (c. 436 G > C) that exchanges a valine for a leucine and one homozygous deletion mutation in *CRYAA* that leads to an in-frame deletion of three amino acids, are likely the dominant cause of cataracts in these two families. Functional analysis showed that the *CRYBB2* mutation appears to abolish βB2-crystallin solubility and stabilization, leading to protein aggregation in human lens epithelial cells, whereas the *CRYAA* deletion mutation causes abnormal protein distribution. Furthermore, our results demonstrate that the *CRYBB2* and *CRYAA* mutations lead to apoptosis in human lens epithelial cells due to UPR. These findings extend the mutation spectrum of crystallin genes in the Chinese CC population and provide clues for exploring the genetic mechanism of CC.

## Results

### Clinical examination

**Figure 1:**
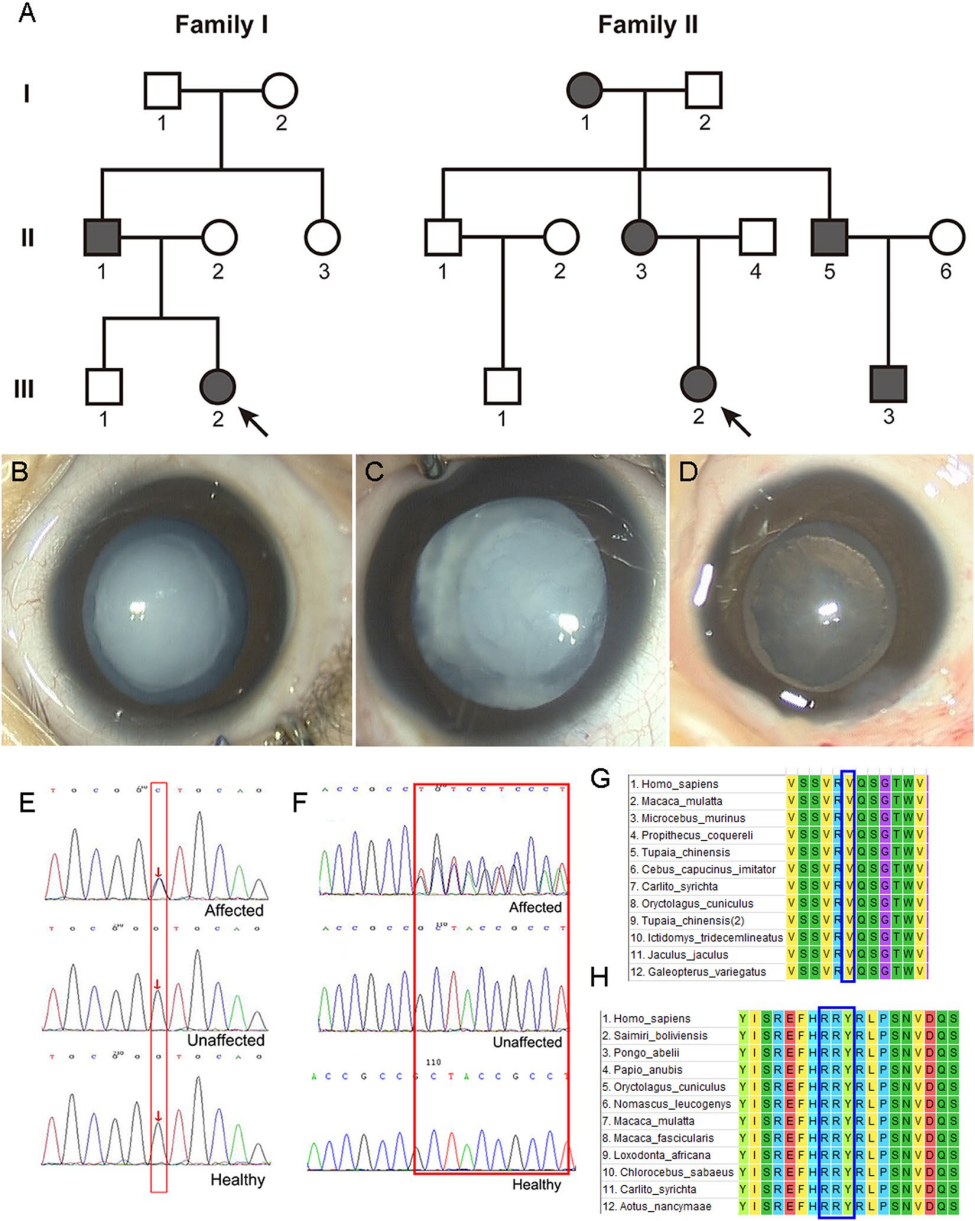
Family pedigree, clinical features, mutation screening and multiple sequence alignment analysis. (**A**) Family history in three-generation families I and II. Dark symbols indicate affected members; clear symbols represent unaffected members. Squares indicate males and circles females. The black arrow represents the proband. (**B**,**C**) Slit-lamp photographs of both eyes retrieved from the proband of Family I. (**D**) Slit-lamp photograph of the affected eye from the proband of Family II. (**E**,**F**) Screening of the mutation sites in the two families by sequence analysis. The red frame represents mutation sites. (**G**,**H**) Multiple sequence alignment of *CRYBB2* and *CRYAA* from different organisms. The blue frame represents mutation sites.

Two Chinese families from Henan Province were enrolled in this study. As shown in Fig. 1A, the pedigrees of the two families revealed an autosomal dominant inheritance pattern. Family I is a three-generation family with two affected members and five unaffected members. According to clinical diagnosis, the four-month-old proband in Family I suffered from a total cataract. As shown in Fig. 1B,C, opacities were observed in the entire lens in both eyes but were more severe in the lens nucleus. Family II was also a three-generation family, including five affected patients and six unaffected individuals. The three-month-old proband was afflicted with a nuclear cataract; the infant suffered from uneven and gray opacification in the lenticular nucleus, though no opacity was found in the peripheral cortex of the lens (Fig. 1D). None of the participating members of both families suffered from other related ophthalmic or systemic syndromes.

### Two novel mutation sites were identified by NGS and Sanger sequencing

Nearly 134 known candidate genes were evaluated by sequencing, and 64 cataract genes were detected by NGS in the probands. Overall, 560.25 Mb of raw data and 548.39 Mb of processed data were retrieved. The mean coverage of the target region was observed to be more than 95%, with an average sequencing depth of >400X. In addition, the coverage of the targeted base for the N10 and N20 reads was 82.9% and 74.3%, respectively. Two variants remained after filtering of existing variants with a minor allele frequency (MAF) greater than 0.05 in databases (dbSNP138, 1000 Genomes, and in-house Asia database).

*In silico* analysis of the NGS data revealed a novel missense and a deletion mutation in *CRYBB2* and *CRYAA*, respectively. In Family I, a missense mutation in the exon of *CRYBB2* (c.436 G > C) that leads to substitution of the conserved valine to a leucine at codon 146 (p.V146L) was identified (Fig. 1E). A heterozygous deletion mutation, c.344_352del, in *CRYAA* that results in an in-frame deletion of three residues from codons 116–118 (p.116_118del) was found in the proband of Family II (Fig. 1F). Moreover, Sanger sequencing analysis revealed the lack of these mutations in all unaffected family members and healthy controls. These results show that the mutations were not observed in unaffected relatives and healthy controls from the same ethnic background.

### Bioinformatic analysis

Four online prediction tools, Mutation Taster, PolyPhen-2, PROVEAN and SIFT, were employed to evaluate the structural and functional effects of the missense mutation p.V146L in βB2-crystallin, and Mutation Taster and PROVEAN were used to assess the *CRYAA* deletion mutation. The *CRYBB2* mutation was predicted to be ‘probably benign’ (score: 0.005), with no damage, according to PolyPhen-2, and PROVEAN predicted a neutral effect. However, the other two predictions indicated that the missense mutation would be deleterious. Similarly, PROVEAN and Mutation Taster predicted the *CRYAA* deletion to be deleterious.

To evaluate the effects of mutations in crystallin, the simulation program SWISS-MODEL was utilized to predict the 3-D structure of both the mutant and wild-type proteins. As shown in Supplementary Fig. S1, we observed a difference between the structure of the mutated and wild-type αA-crystallin protein, which in turn resulted in structural variation. In contrast, the missense mutation in βB2-crystallin appears to have a lower structural impact.

Furthermore, multiple sequence alignment was performed to explore the conserved nature of these mutations. As shown in Fig. 1G, a valine at the 146^th^ position of βB2-crystallin is highly conserved among several mammalian species. Arginine-arginine-tyrosine are also highly conserved in most of the species examined (Fig. 1H).

These results demonstrate that the missense p.V146L and the deletion p.116_118del might be deleterious mutations, resulting in CC.

### Functional verification of transfected cells

**Figure 2:**
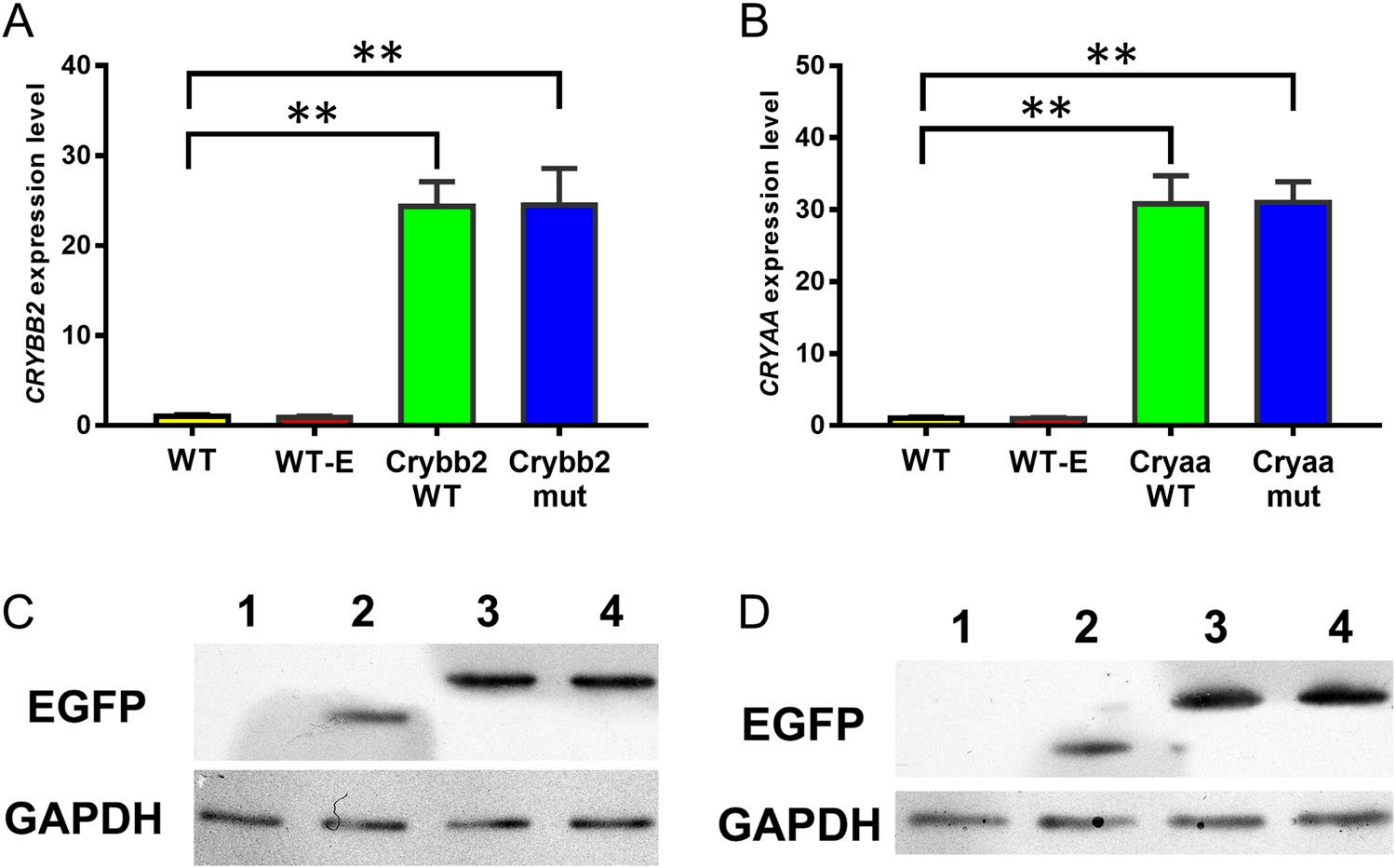
Molecular verification of transfected cells. (**A**) *CRYBB2* relative expression level in untransfected and transfected cells. WT, wild-type cells; WT-E, cells expressing EGFP; Crybb2-WT, cells expressing normal *CRYBB2*; Crybb2-mut, cells expressing mutant *CRYBB2*. (**B**) *CRYAA* relative expression level in normal cells and transfected cells. WT, wild-type cells; WT-E, cells expressing *EGFP*; Cryaa-WT, cells expressing normal *CRYAA*; Cryaa-mut, cells expressing mutant *CRYAA*. *GAPDH* was used as a housekeeping gene. (**C**) Western blot analysis of βB2-crystallin in normal cells and transfected cells. 1, wild-type cells; 2, cells expressing EGFP; 3, cells expressing normal βB2-crystallin; 4, cells expressing mutant βB2-crystallin; GAPDH was used as the internal control. The original western blot images are shown in Supplementary Fig. S2 and S3. (**C**) Western blot analysis of αA-crystallin in normal cells and transfected cells. 1, wild-type cells; 2, cells expressing EGFP; 3, cells expressing normal αA-crystallin; 4, cells expressing mutant αA-crystallin, GAPDH was used as the internal control. The original western blot images are shown in Supplementary Fig. S4 and S5.

Transfected HLEpiCs were grown in DMEM supplemented with G418. *CRYBB2* and *CRYAA* mRNA was assessed by quantitative polymerase chain reaction (qPCR) analysis, which revealed a significantly higher transcript abundance than in non-transfected and enhanced green fluorescent protein (EGFP)-transfected cells (Fig. 2A,B). Expression of the proteins was further evaluated by western blotting using an anti-EGFP antibody. As shown in Fig. 2,D, a specific cross-reactive band was observed in transfected cells, whereas no such band was detected in non-transfected cells. These results reveal that both genes, with or without mutation, were successfully expressed in transfected cells.

### Fluorescence observation of protein distribution and aggregation

**Figure 3:**
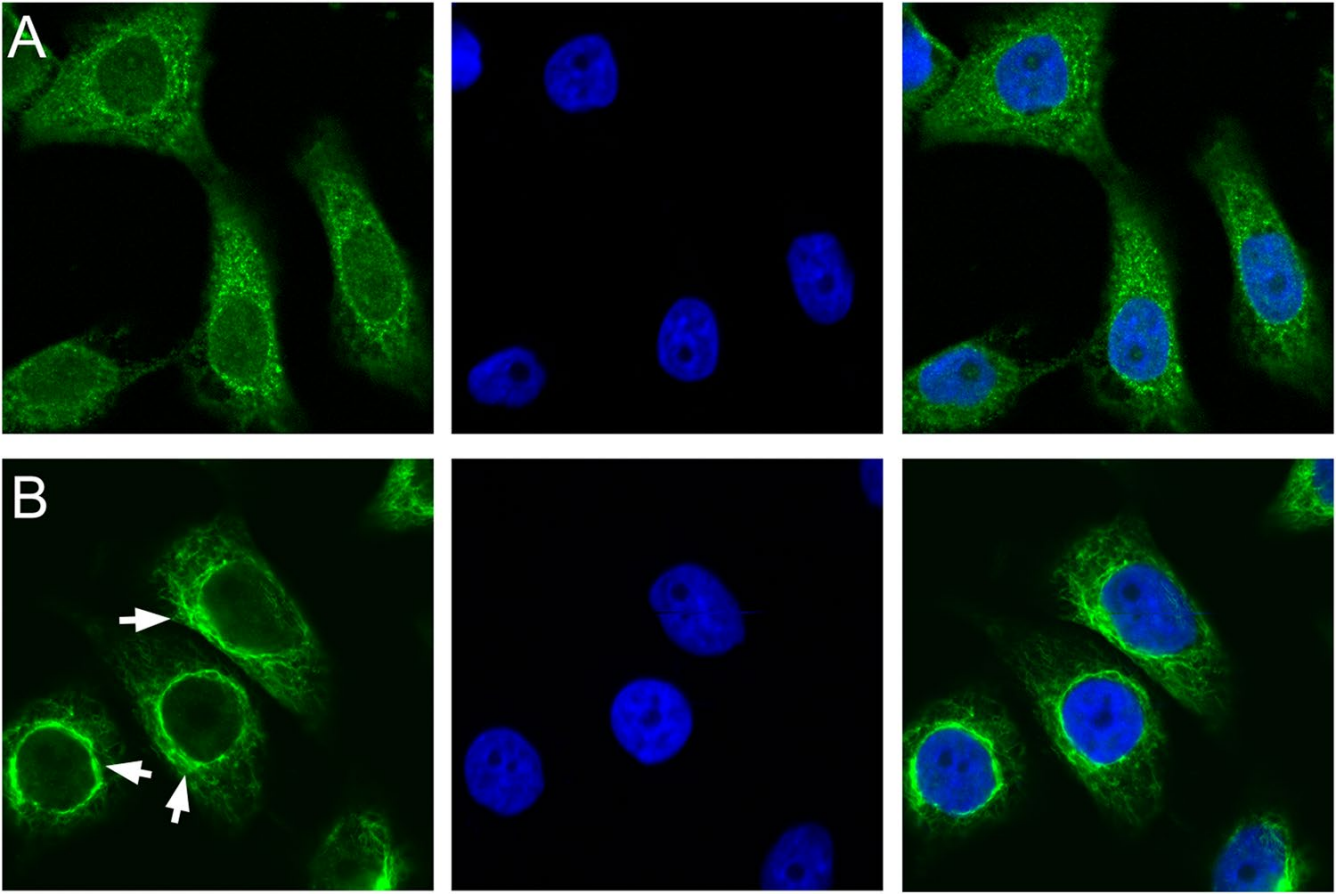
Representative fluorescence microscopy images of HLEpiCs expressing wild-type βB2-crystallin fused to EGFP or mutant βB2-crystallin fused to GFP. (**A**) Cells expressing wild-type βB2-crystallin displayed hypodispersion. (**B**) Mutant βB2-crystallin aggregated at the nuclear peripheral membrane. The white arrow indicates the location of protein aggregation. Left, GFP fluorescence; middle, DAPI (4′,6-diamidino-2-phenylindole) nuclear fluorescence; right, overlay.

**Figure 4:**
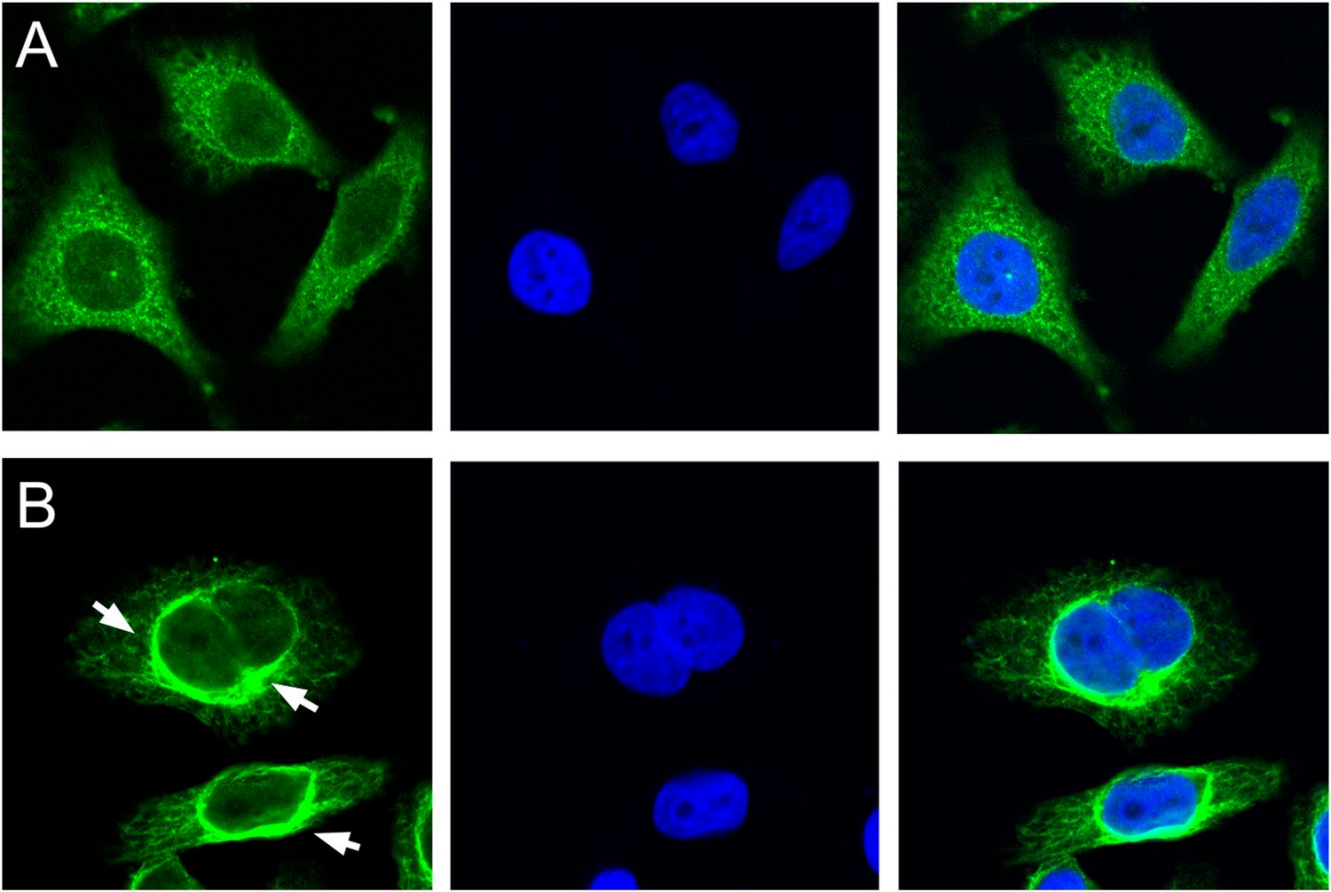
Representative fluorescence microscopy images of HLEpiCs expressing wild-type αA-crystallin fused to GFP or mutant αA-crystallin fused to GFP. (**A**) Cells expressing wild-type αA-crystallin displayed hypodispersion; (**B**) Mutant αA-crystallin aggregated at the nuclear peripheral membrane. The white arrow indicates the location of protein aggregation. Left, GFP fluorescence; middle, DAPI (4′,6-diamidino-2-phenylindole) nuclear fluorescence; right, overlay.

Fluorescence observation was used to determine the impact of the mutant proteins in HLEpiCs. As shown in Fig. 3, normal βB2-crystallin was detected and uniformly distributed in the cytoplasm, which is consistent with the results of previous studies. In contrast, βB2-crystallin-V146L was observed to be aggregated in the nuclear peripheral membrane. Wild-type αA-crystallin was also equally distributed in the cytoplasm, whereas the deletion mutation accumulated at the nuclear peripheral membrane, with less inside the nucleus (Fig. 4). These results indicate that the p.V146L mutation in βB2-crystallin and p.116_118del in αA-crystallin disrupt protein transport and localization and promote protein aggregation at target sites.

### Mutant proteins influence apoptosis

**Figure 5:**
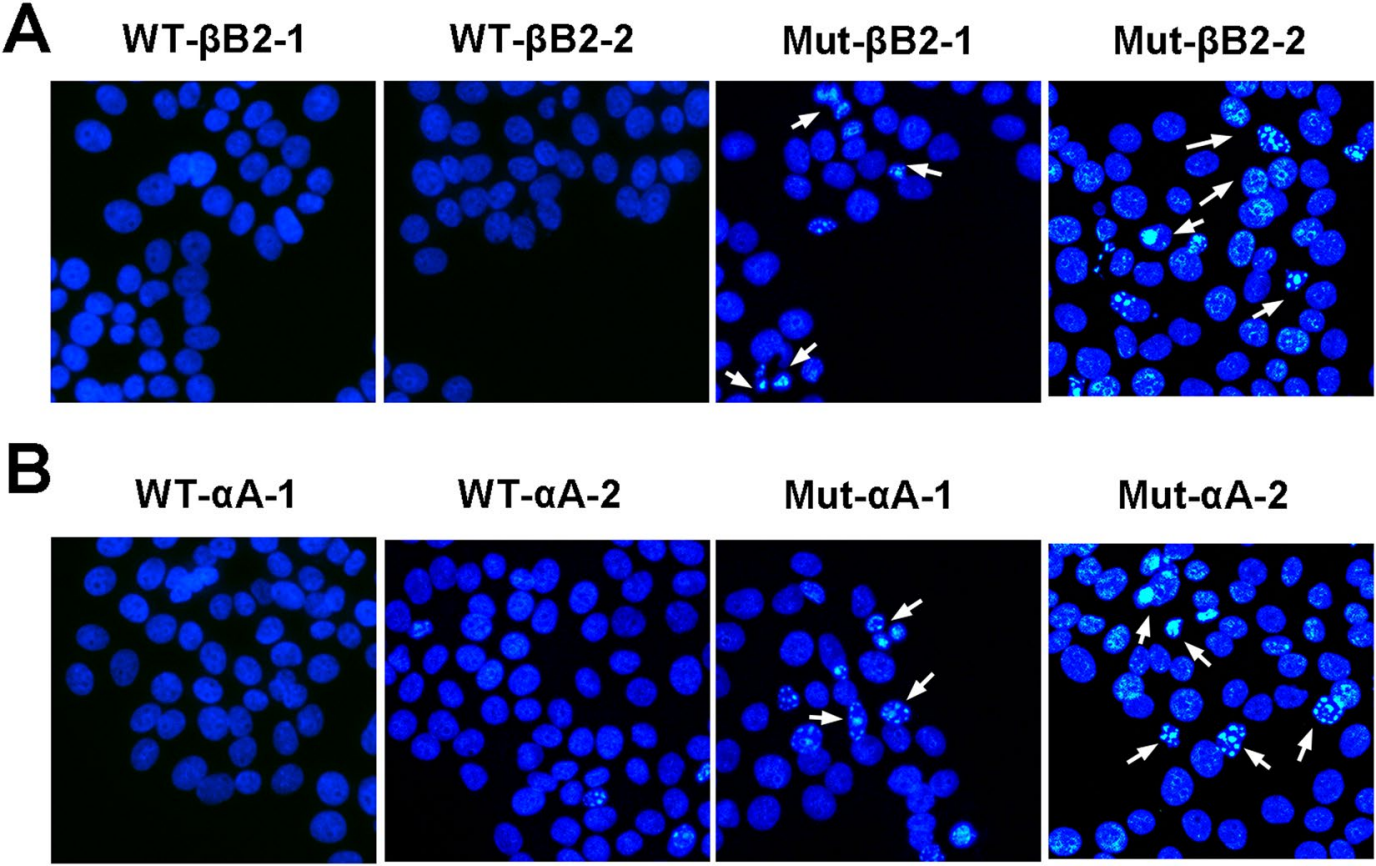
Representative fluorescence microscopy images of HLEpiCs expressing wild-type βB2-crystallin or αA-crystallin or mutants. (**A**) Cells expressing wild-type βB2-crystallin and mutant βB2-crystallin. (**B**) Cells expressing wild-type αA-crystallin and mutant αA-crystallin. The white arrow indicates an apoptotic cell.

We used Hoechst 33342 staining to determine the influence of the mutations on apoptosis. As shown in Fig. 5, overexpression of wild-type βB2-crystallin in HLEpiCs did not affect viability. Conversely, mutant βB2-crystallin accelerated apoptosis (Fig. 5A), as did mutant αA-crystallin (Fig. 5B).

**Figure 6:**
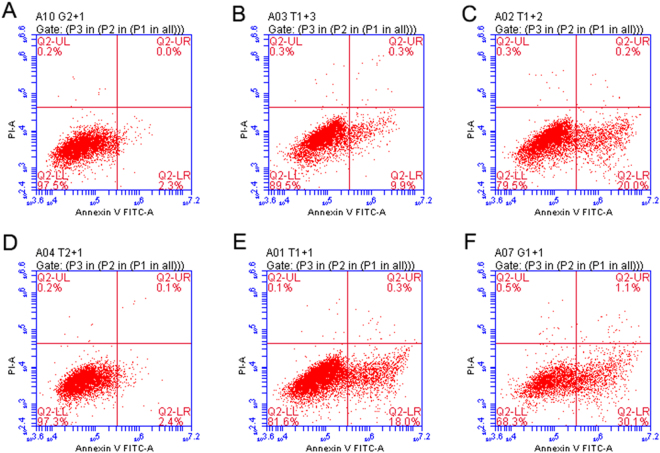
Apoptotic cells expressing wild-type and mutant crystallins according to flow cytometry (**A**–**C**) Non-transfected cells and those expressing wild-type βB2-crystallin and mutant βB2-crystallin, respectively. (**B**) Non-transfected cells and those expressing wild-type αA-crystallin and mutant αA-crystallin.

Furthermore, we also assessed apoptosis by Annexin V-fluorescein isothiocyanate (FITC)/propidium iodide (PI) staining, whereby the increment in Annexin V-positive/PI-negative cells reflect increased apoptosis. As shown in Fig. 6A–C, cells expressing mutant βB2-crystallin displayed a significant increase in apoptosis compared to cells expressing wild-type βB2-crystallin and control cells. Cells overexpressing mutant αA-crystallin also exhibited more apoptosis compared to cells expressing wild-type αA-crystallin and control cells (Fig. 6D–F). These results demonstrate that the βB2-crystallin p.V146L and αA-crystallin p.116_118del mutations potently induce apoptosis.

**Figure 7:**
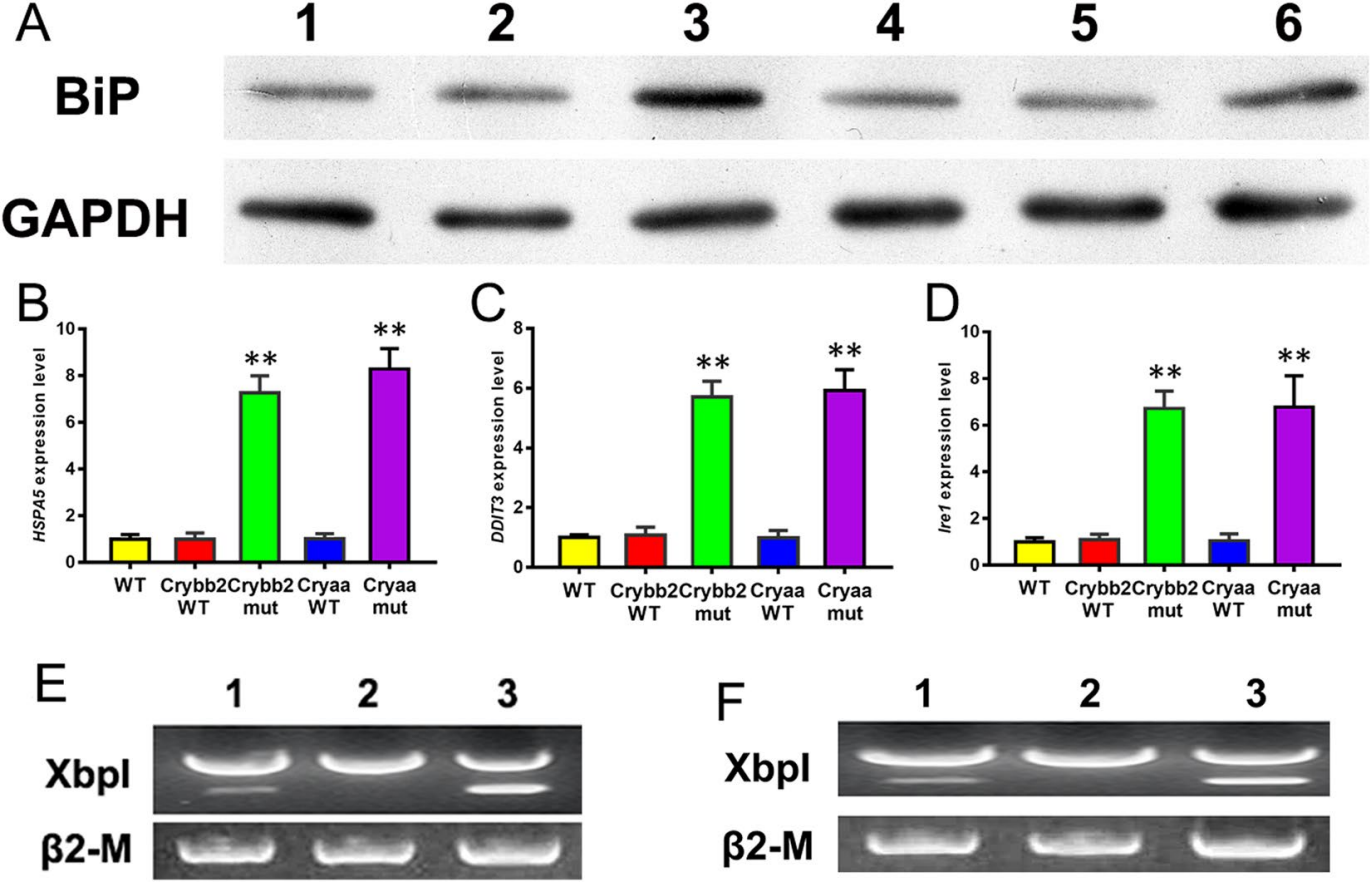
UPR-associated gene expression status in cells. (**A**) Western blotting analysis confirming the increase in BiP expression in cells expressing the βB2-crystallin and αA-crystallin mutants. 1, cells without transfection; 2, cells expressing wild-type βB2-crystallin; 3, cells expressing mutant βB2-crystallin; 4, cells without transfection; 5, cells expressing wild-type αA-crystallin; 6, cells expressing mutant αA-crystallin. GAPDH was used as an internal control gene. The original western blot image is shown in Supplementary Fig. S6 and S7. (**B**–**D**) HSPA5, DDIT3 and Ire1 relative expression levels in normal cells and transfected cells. WT, wild-type cells; Cryaa-WT, cells expressing normal cryaa; Cryaa-mut, cells expressing mutant cryaa; Cryaa-WT, cells expressing normal cryaa; Cryaa-mut, cells expressing mutant cryaa. GAPDH was used as a housekeeping gene. (**E**,**F**) Xbp1 splicing detection. 1, wild-type cells; 2, cells expressing wild-type crystallin; 3, cells expressing mutant crystallin.

Previous studies reported that UPR is important in the pathogenesis of protein aggregation in cataracts^16,29,30^. To determine the apoptosis mechanism induced by the crystallin mutations, we assessed expression of BiP, an endoplasmic reticulum (ER) protein that strongly promotes UPR activation, in both mutant transfected cells. As shown in Fig. 7A, western blot analysis revealed the BiP expression level to be upregulated in cells expressing both mutants. Transcription of UPR genes, including HSPA5 and DDIT3, was also markedly increased (Fig. 7B,C). During UPR activation, the transmembrane sensor Ire1 is first activated via non-canonical splicing of X-box binding protein 1 (Xbp1) mRNA, and the level of Ire1 transcription was extremely elevated in both mutants compared to cells expressing wild-type crystallin and control cells (Fig. 7D). In addition, as shown in Fig. 7E,F, the level of spliced Xbp1 was higher in cells expressing mutants.

## Discussion

CC is a serious hereditary disease resulting in blindness with clinical and genetic heterogeneity, and autosomal dominant inheritance is reported to be the major cause of CC. With the advent of high-throughput molecular techniques, more research is being conducted on the genetic basis of CC. Shiels *et al*. reported mutation in crystallin genes among nearly half of CC cases^31^. In this study, we identified two novel mutations in two three-generation Chinese families by NGS and reconfirmed these results by Sanger sequencing.

Crystallins are the predominant lens structural proteins of mammals and have a significant function in providing transparency and light transmission to the eye lens^32^. However, mutations in the genes encoding crystallin proteins lead to abnormal expression, which might disrupt lens opacity and even cause blindness^12,33,34^.

Previous studies have demonstrated that expression of *CRYAA* is necessary for normal lens development and that knockout of α-crystallins resulted in abnormal differentiation of lens fiber cells in zebrafish^35^. Similarly, transgenic expression of a mutant crystallin (*CRYBA1*) protein resulted in abnormal differentiation of lens fibers in transgenic mice and consequently led to lens capsule rupture^26^. Hence, it is clear that mutated crystallin proteins have a pivotal role in lens development in animal cells; however, there are very few studies reporting the type of mutation in these genes and impacts on lens differentiation in human lens cell lines. The protein αA-crystallin is responsible for preventing apoptosis through chaperone-analogous activities and for protecting lens proteins from precipitation^36,37^. *CRYAA*-encoded αA-crystallin has been mapped to chromosome 21q22.3, and several mutations in the *CRYAA* gene have been associated with various CC types worldwide^14,38,39^. For instance, the missense mutation p.R116C causes a decrease in positive charge and an increase in mercapto groups in αA-crystallin, leading to polymer hydrophobicity and protein precipitation^38^. Missense mutations of p.R116H and p.G98R reduce the stability of αA-crystallin, which, in turn, causes cataracts^13,15^. Notably, in this study, we identified a c.344_352del in-frame mutation in *CRYAA* that causes deletion of three amino acids (residues 116–118), and this mutation results in significant variation in protein distribution, with notable aggregation at the nuclear peripheral membrane and nucleus based on fluorescence observation (Fig. 4B).

βB2-crystallin encoded by *CRYBB2* contains four key Greek sequences; the six exons of *CRYBB2* map to chromosome 22q11.2-q13.1. The first two Greek key motifs are in the N-terminal domain, and the other two are located at the COOH-terminal domain. The solubility and stability of βB2-crystallin are crucial for proper function in the lens, with disruption of solubility and stability causing aggregation, which leads to lenticular transparency and diopter damage^40^. Previous studies have reported that mutations in *CRYBB2* are associated with the onset of various types of CC. For example, a missense mutation (p.A2V) in the N-terminal domain influences the renaturation process, resulting in βB2-crystallin aggregation^41^. At the C-terminal domain, the p.Q155X nonsense mutation impacts formation of the Greek key motif, affecting folding and biophysical properties that lead to congenital cataract^42^. In the present study study, it was observed that the p.V146L mutation alters the molecular weight and hydrophobic properties of βB2-crystallin. Previous studies showed the significance of βB2-crystallin in β-crystallin aggregation within various contexts^43,44^. Consistently, our overexpression analysis of the mutant βB2-crystallin in cell lines revealed aggregation of the protein at the nuclear peripheral membrane (Fig. 3B). Altogether, the reported missense and deletion mutations in *CRYBB2* and *CRYAA* might be common and responsible for crystallin protein aggregation.

Our results showed that mutations in *CRYAA* and *CRYBB2* induce expression of UPR-associated genes, such as HSPA5, DDIT3 and Ire1. UPR is an adaptive intracellular signaling mechanism that responds to the accumulation of misfolded proteins by triggering upregulation of a characteristic group of target genes^26,45–47^. Earlier studies have reported a crucial role for UPR genes, such as HSPA5, DDIT3 and Ire1, in response to misfolded proteins^45,48,49^. HSPA5 has been considered to be the key protein in UPR, as it has dual functions as an ER chaperone and as a sensor of protein misfolding^50^. In our study, we observed that expression of UPR-associated genes was significantly increased in transgenic cells expressing mutant αA-crystallin and βB2-crystallin (Fig. 7) and the occurrence of apoptosis in cells expressing these mutants (Figs 5 and 6). Expression of mutant G98R αA-crystallin in human epithelial B3 cells induced UPR-mediated apoptosis^51^. Firtina *et al*. demonstrated that abnormal expression of collagen IV genes triggers transcriptional activation of UPR genes and consequently induces apoptosis and cataract formation in transgenic fiber cells^52^. Congruently, higher expression of UPR-specific proteins induced apoptosis in lens epithelial cells as well as cataract formation in rats^30^. In addition, mutant αA-crystallin R49C triggered UPR, which induced lens cell death in a mutant knock-in mouse model^16^. Thus, mutant αA-crystallin and βB2-crystallin cause upregulation of characteristic UPR genes and, consequently, apoptosis in transfected cells, which is in accordance with previous reports. In conclusion, two new heterozygous mutations (p.116_118del in *CRYAA* and p.V146L in *CRYBB2*) were identified in two Chinese families with ADCC. The deletion mutation p.116_118del was found to influence protein aggregation, and the missense mutation p.V146L was determined to affect protein distribution and induce apoptosis in HLEpiCs. Our findings provide some clues for the vital role of crystallin and the mechanism by which mutation results in CC. Moreover, our results also extend the mutation spectrum of CC causative genes in the Chinese population.

## Methods

### Ethics statement

This study was performed in adherence with the Declaration of Helsinki and was approved by the Institutional Review Board of the First Affiliated Hospital at Zhengzhou University (Zhengzhou, China). Written informed consent was obtained from all participants (or their legal guardians).

### Subjects, clinical examination and DNA isolation

Two Chinese families of the Han ethnicity from Henan Province affected by CC were recruited from the ophthalmology department at First Affiliated Hospital of Zhengzhou University. One hundred unrelated participants without eye disease were also enrolled from the ophthalmology department as healthy controls. Family and medical history were recorded. Complete ophthalmic examinations, including visual acuity, dilated pupil examination, intraocular pressure measurement, and slit-lamp ophthalmoscopy, were performed. The phenotypes obtained by slit-lamp photography were documented. A 5-mL sample of venous blood was obtained from all participants and placed in tubes containing ethylenediaminetetraacetic acid (EDTA). Genomic DNA was extracted using QIAamp DNA Blood Mini Kit (Qiagen, USA) according to the manufacturer’s recommendations.

### NGS and Sanger sequencing to validate variants

All candidate genes (134 genes, including 64 cataract genes) implicated in CC were subjected to sequencing. The 64 cataract genes are listed in Supplementary Dataset 1. Libraries were prepared following standard protocols, as previously described^53,54^. Targeted sequence capture was performed with biotinylated oligo-probes and a disease-related gene panel following the manufacturer’s instructions. Paired-end sequencing for reads of 100 bp was performed using the Illumina HiSeq. 2000 platform (Illumina, USA).

Low-quality sequences in the raw reads were filtered by Trim-Galore, and clean reads were then aligned to the human reference genome using the BWA program. Quality scores of the clean reads were recalibrated and realigned for reference with GATK software. Sequence Alignment/Map tools 3 (SAMtools 3) were used for removing duplicated reads, and only unique mapping reads were further applied for variation detection. Single-nucleotide variants were analyzed and genotyped by GATK UnifiedGenotyper, and indels were analyzed by GATK Indel GenotyperV2. Variants were annotated by an in-house bioinformatic tool with RefSeq (hg19, from UCSC) and UCSC annotation according to the manufacturer’s recommendations.

Two genes acquired by NGS were defined as probable causative genes, and Sanger sequencing was performed for validation. PCR products with mutation were amplified using primers and purified with a gel extraction kit (Omega, USA). An ABI DNA Analyzer (Applied Biosystems, USA) was used to analyze the sequence data. Damage predictions were performed using bioinformatic tools such as PROVEAN, MutationTasting, SIFT and PolyPhen-2^55–58^.

### Cell culture and transfection

Human lens epithelial cells (HLEpiCs) were purchased from American Type Culture Collection (ATCC) and cultured in Dulbecco’s modified Eagle’s medium (DMEM, Gibco, USA) fortified with 10% fetal bovine serum (FBS, Gibco, USA). The cells were cultivated at 37 °C in a humidified atmosphere containing 5% CO_2_. Plasmids carrying normal or mutant genes were constructed by restriction enzyme ligation and transfected into cells using Lipofectamine 2000 (Invitrogen, USA) following the manufacturer’s protocols.

### Molecular characterizations of transfected cells

To determine the relative expression of mutant and non-mutant *CRYBB2* and *CRYAA* in transfected cells, quantitative real-time PCR (RT-qPCR) was performed following the protocol described by Wang *et al*.^59^. Efficiency was determined before performing qPCR, and only primers with an efficiency above 95% were used. Endogenous GAPDH was used for normalization of the threshold cycle (Ct) value detected for both the transfected and normal cells. Western blotting was also carried out to verify expression of the proteins in the transfected cells. Cells transfected with GFP served as controls. Target proteins were blotted using a primary anti-GFP antibody (1:20000, Abcam, USA) and horseradish peroxidase (HRP) Goat anti-Rabbit IgG antibody (1:20000, Boster, USA). Protein expression was normalized to that of GAPDH.

### Fluorescence microscopy for determination of aggregation

HLEpiCs transfected for 48 h were used for fluorescence microscopy to observe protein distribution. Briefly, cells were washed three times with phosphate-buffered saline (PBS) and fixed with acetone for 10 min at room temperature. The fixed cells were washed three times with PBS and then stained with DAPI for 5 min at room temperature. The cells were observed by fluorescence microscopy. The percentage of cells with aggregates was calculated from 200 positively transfected cells in 10 random viewing fields.

### Apoptosis determination

Apoptosis caused by the mutations was evaluated by nuclear morphology observation of Hoechst 33342-stained cells. In brief, transfected cells were cultivated in 6-well plates and stained with Hoechst 33342, after which the cells were observed by fluorescence microscopy. The numbers of apoptotic nuclei were counted in five random viewing fields.

Transfected cells were first stimulated with 400 μM H_2_O_2_ for 24 h, and an apoptosis assay was then performed using Annexin V-FITC/PI Apoptosis Detection Kit (Vazyme, China) according to the manufacturer’s instructions. The cells were assessed by flow cytometry using a BD FACS Aria-IIu apparatus and Cell Quest software (BD Biosciences, USA).

### Molecular analysis of apoptosis induced by mutant crystallin

Quantitative real-time PCR was performed as described above. The ribosomal protein gene Rpl19 served as an endogenous control for quantitation of UPR-related genes. For RT-PCR, total RNA was extracted from transfected cells using a Total RNA kit (Omega, USA). Each reaction used 20 ng of total RNA as a template, and the primers used were selected according to a previous study^52^. Western blot analysis was also performed according to a previous study^52^, and a rabbit polyclonal antibody against BiP/Grp78 (Abcam, UK) was used as the primary antibody.

## Electronic supplementary material

Supplementary File 1

Supplementary Dataset 1
