# Supplementary material for: Two novel mutations identified in ADCC families impair crystallin protein distribution and induce apoptosis in human lens epithelial cells
Source: Sci Rep. 2017 Dec 19;7:17848. doi: 10.1038/s41598-017-18222-z (PMC5736644; doi:10.1038/s41598-017-18222-z)

**Two novel mutations identified in ADCC families impairs** **crystallin protein distribution and induces apoptosis of human lens epithelial cells**

Li Li^+1,2^, Da-Bei Fan^+3^, Ya-Ting Zhao^1,2^, Yun Li^1,2^, De-Qian Kong^1,2^, Fang-Fei Cai^1,2^, Guang-Ying Zheng*^1,2^

**Supplementary Fig. S1** 3-D structure of both the mutant and wild CRYBB2 and CRYAA protein predicted by SWISS-MODEL. (A) 3-D structure of wild CRYBB2. (B) 3-D structure of mutant CRYBB2. (C) 3-D structure of wild CRYAA. (D) 3-D structure of mutant CRYAA. Red circle indicates mutation site.


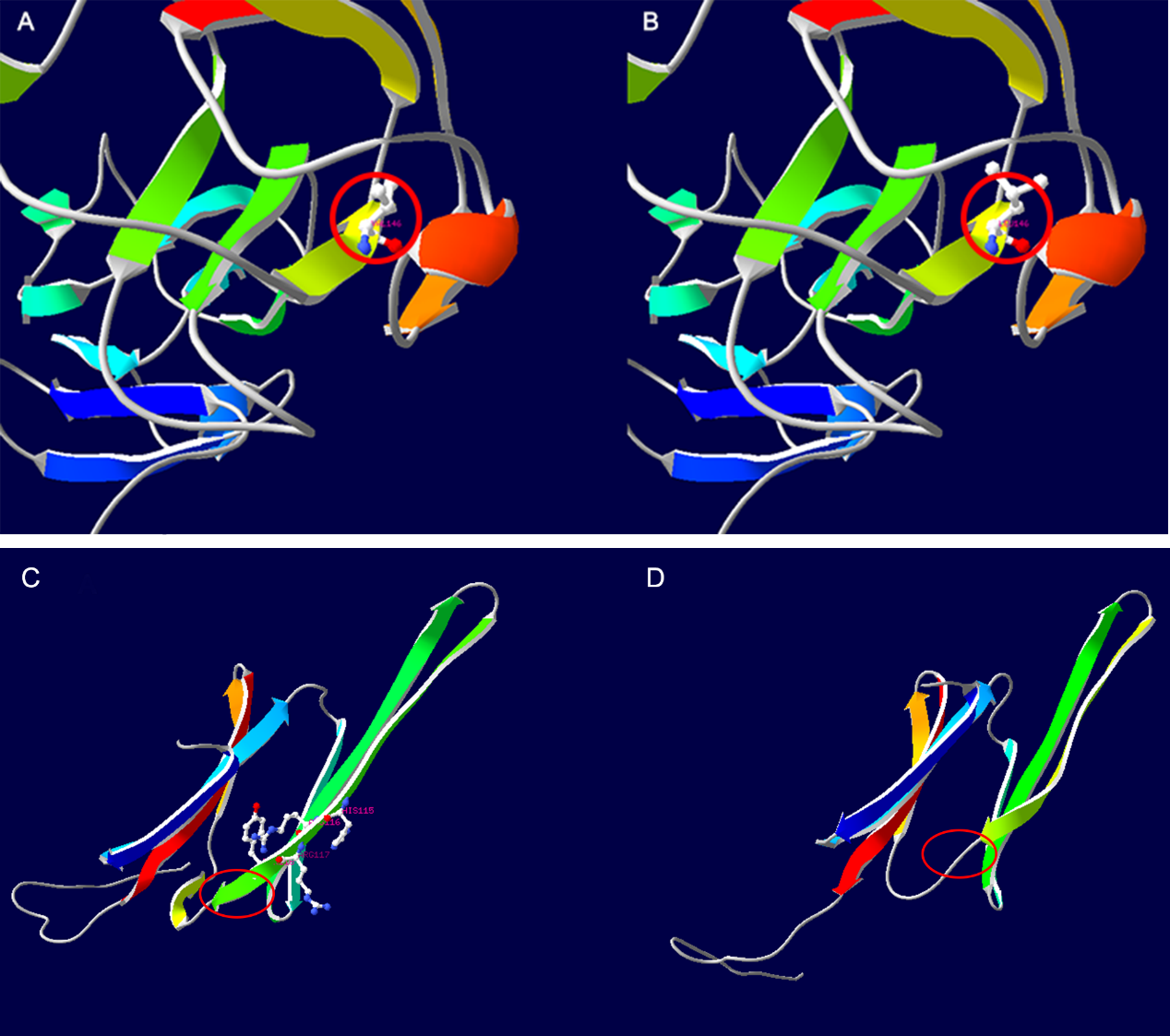


**Supplementary Fig. S2** Original western blot digital image of proteins against primary antibody anti-EGFP. From left to right: cells without transfection; cells transfected with eGFP vector; cells transfected with wild CRYBB2; cells transfected with mutant CRYBB2.


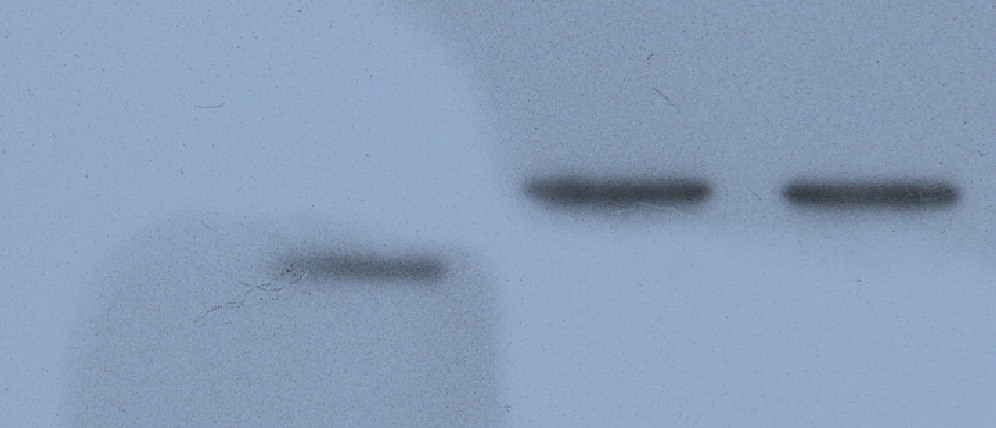


**Supplementary Fig. S3** Original western blot digital image of proteins against primary antibody anti-GAPDH. From left to right: cells without transfection; cells transfected with eGFP vector; cells transfected with wild CRYBB2; cells transfected with mutant CRYBB2.


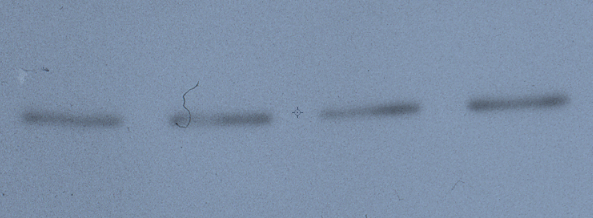


**Supplementary Fig. S4** Original western blot digital image of proteins against primary antibody anti-EGFP. From left to right: cells without transfection; cells transfected with eGFP vector; cells transfected with wild CRYAA; cells transfected with mutant CRYAA.


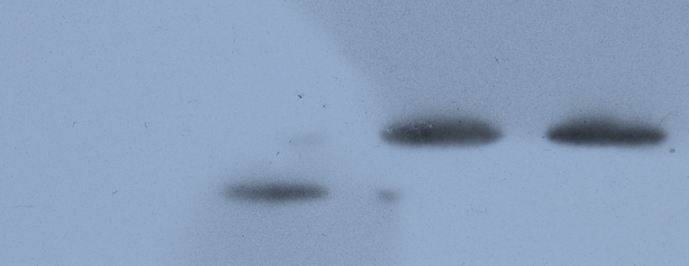


**Supplementary Fig. S5** Original western blot digital image of proteins against primary antibody anti-GAPDH. From left to right: cells without transfection; cells transfected with eGFP vector; cells transfected with wild CRYAA; cells transfected with mutant CRYAA.


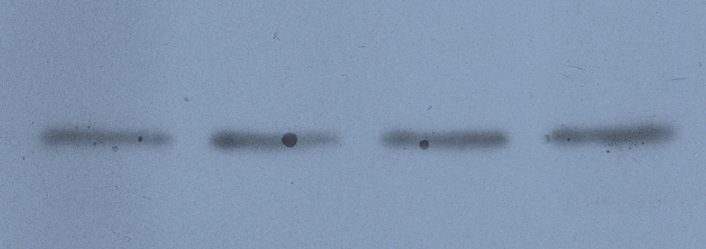


**Supplementary Fig. S6** Original western blot digital image of proteins against primary antibody anti-BiP/Grp78. From left to right: Cells transfected with eGFP vector; cells transfected with wild CRYAA; cells transfected with mutant CRYAA; cells transfected with eGFP vector; cells transfected with wild CRYBB2; cells transfected with mutant CRYBB2.


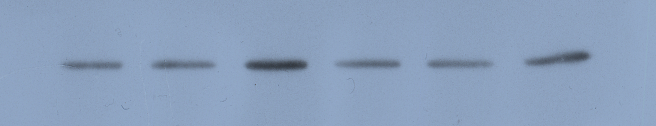


**Supplementary Fig. S7** Original western blot digital image of proteins against primary antibody anti-GAPDH. From left to right: Cells transfected with eGFP vector; cells transfected with wild CRYAA; cells transfected with mutant CRYAA; cells transfected with eGFP vector; cells transfected with wild CRYBB2; cells transfected with mutant CRYBB2.


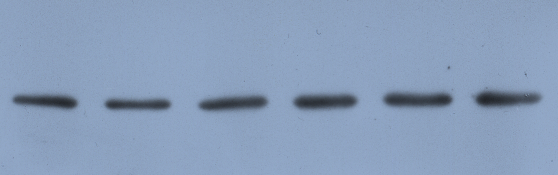

Supplement: Supplementary file 1 [file 41598_2017_18222_MOESM1_ESM.docx]
